# Influence of Ongoing Task Difficulty and Motivation Level on Children’s Prospective Memory in a Chinese Sample

**DOI:** 10.3389/fpsyg.2017.00089

**Published:** 2017-02-01

**Authors:** Pi-guo Han, Lei Han, Yu-long Bian, Yu Tian, Min-xia Xu, Feng-qiang Gao

**Affiliations:** ^1^School of Psychology, Shandong Normal UniversityJinan, China; ^2^Department of Preschool Education, Heze UniversityHeze, China; ^3^Department of Computer Science and Technology, Shandong UniversityJinan, China

**Keywords:** prospective memory, ongoing task difficulty, delayed retrieval, event-based, time-based, motivation

## Abstract

Prospective memory (PM) is the process associated with the task of realizing delayed intentions in the future. Researchers distinguish two types of PM, namely time-based PM (tbPM) and event-based PM (ebPM). Experiment 1 investigated the developmental trajectory of 3- to 5-year-old preschool children’s PM ability, and the occurrence of delayed retrieval (children execute the PM task in a larger window of opportunity) in both tbPM and ebPM tasks. Results revealed that the 5-year-old children outperformed the 3- and 4-year-old children in PM. Moreover, delayed retrieval was more likely to occur in tbPM task than in ebPM task. In Experiment 2, the influence of ongoing task (OT) difficulty on PM performance was investigated with a sample of 5-year-old children. Results revealed no significant effect of OT difficulty on PM performance. In Experiment 3, we improved children’s motivation level to complete the OT, then explored the influence of OT difficulty on children’s PM performance. Results revealed that the effect of OT difficulty on PM performance became significant after increasing the children’s motivation to complete the OT. These results provide insights into the mechanism of attentional resource allocation in PM tasks and have crucial educational and social implications.

## Introduction

Prospective memory (PM) is the process associated with the task of realizing delayed intentions in the future (Einstein and McDaniel, 1990; Einstein et al., 1992; Ford et al., 2012). Researchers distinguish two types of PM, namely time-based PM (tbPM) and event-based PM (ebPM) (Kvavilashvili and Ellis, 1996; Delprado and Kinsella, 2013; Li, 2014; Talbot and Kerns, 2014). In tbPM tasks, intentions are executed at a specific time-point in the future or after a definite time span has elapsed (Wang et al., 2008); for example, remembering to call a friend on their birthday or remembering to take medicine after half an hour. In ebPM tasks, the appropriate moment for task execution is indicated by an external cue (Talbot and Kerns, 2014) such as remembering to give friends a message upon next encountering them. PM forms an integral part of everyday life for all age groups (Talbot and Kerns, 2014). In daily life, young children often must complete PM tasks such as remembering to tell their parents to sign their homework when they arrive home from school, or to take something to kindergarten the next day (Kretschmer et al., 2014). Children with poor PM ability are likely to experience difficulties when interacting with parents, teachers, and peers (McCauley and Levine, 2004; Kayla and David, 2014). The successful development of PM during childhood is necessary for independent living later in life (Kliegel and Martin, 2003; Wang et al., 2006, 2008). Thus, interest in the development of PM has increased within the past decade (Kvavilashvili et al., 2008) in recognition of its critical educational and social implications (Kayla and David, 2014).

### Developmental Trajectory of Children’s PM Ability

Previous studies have indicated that PM follows a clear developmental trajectory (e.g., Kerns, 2000; Shum et al., 2008; Mackinlay et al., 2009; Rendell et al., 2009; Zinke et al., 2010) and that early childhood is a crucial period for the emergence and development of PM ability (Guajardo and Best, 2000; Kliegel and Jäger, 2007; Kretschmer et al., 2014). For example, Kvavilashvili et al. (2001) found that children as young as 3 years old were capable of performing simple ebPM tasks, whereas Rendell et al. (2009) and Aberle and Kliegel (2010) have reported that children as young as 5 years old were capable of performing both ebPM and tbPM tasks to an extent. Guajardo and Best (2000) reported that the PM performance of 5-year-old children is significantly higher than that of 3-year-old children. Kliegel and Jäger (2007) also observed that the PM performance of 4- to 6- year-old children was significantly higher than that of 3-year-old children.

Although various studies have revealed that PM ability begins to develop in early childhood, the PM performance of school-aged children and adults is nevertheless considerably higher than that of preschool children. For example, studies have indicated that preschool children have limited capability to perform PM tasks (Kvavilashvili et al., 2001; Rendell et al., 2009; Aberle and Kliegel, 2010), whereas typically developing 13-year-olds have been found to be capable of successfully performing PM tasks without difficulty (Kerns, 2000; Kliegel et al., 2008).

### Delayed Retrieval in Children’s PM

Some PM tasks cannot be executed immediately in real-world situations. In standard experimental designs, PM tasks generally have to be performed at a specific time-point in the future. However, this does not resemble some real-world PM tasks. Because of ongoing task (OT) demands, some PM tasks cannot be executed immediately, but must be delayed (Einstein et al., 2000). For example, if parents require children to deliver a message to a teacher, but the teacher is currently busy, the child must wait for the teacher to finish before performing the PM task. Hence, Einstein et al. (2000) proposed the “delay–execute paradigm,” which adds an additional delay after the PM target appears to assess the impact of such delays on PM performance. Rendell et al. (2009) applied the delay-execute paradigm to 60 children with a mean age of 5.2 years. In this study, the PM task involved children being required to remember to press a button to refuel the vehicle while playing a computer driving game (OT). The study assessed their ability to carry out the PM tasks either immediately a target cue appeared or after an additional delay. The results demonstrated that their PM performance decreased under the delay-execute condition.

In addition, in real-word situations, some PM tasks do not necessarily need to be executed at a specific time-point. Both the specific time-point (immediate retrieval) and the time span following the time-point (delayed retrieval) can be considered to be effective periods for the execution of a PM task (Han, 2012). For example, if a teacher asks children to put something in their schoolbags when they arrive at home and to bring it to kindergarten the next day, the children can complete the PM task as soon as they arrive home, or during the interval between arriving at home and leaving for school the following morning. This process is termed the delayed retrieval of a PM task. Han (2012) investigated the occurrence of delayed retrieval in PM tasks in a sample of 3- to 5-year-old Chinese children. In this study, the children had to execute a tbPM (reminding the tester as soon as an hourglass ran out) or ebPM task (picking out a specific picture) while they engaged in an OT (a picture-naming task). The results revealed that the children were able to perform better in the tbPM task when they had the opportunity to execute this task later (i.e., delayed retrieval).

Therefore, valid retrieval in PM tasks includes immediate and delayed retrieval. Immediate retrieval refers to executing a PM task only at a targeted time-point or emerging target event, whereas delayed retrieval refers to executing a PM task during a certain time span after the targeted time-point or emerging target event.

Several researchers have suggested that being able to estimate the passage of time is crucial for tbPM to be successful executed (Kerns, 2000; Aberle and Kliegel, 2010; Zinke et al., 2010). However, the time perception ability of children has not fully developed in early childhood (Luo, 2011; Sylvie, 2012); therefore, delayed retrieval is likely a critical PM retrieval mode in early childhood. Moreover, the factors influencing PM may differ between preschool children and other age groups (Meeks et al., 2007; Penningroth and Scott, 2013). However, few studies have investigated these issues. Hence, applying delayed retrieval conditions may enable us to determine the factors influencing PM in children and thereby increase our knowledge of the mechanisms involved in attentional resource allocation.

### Influence of OT Difficulty and Motivation Level on PM Performance

An essential feature of PM tasks is that they have to be performed while an individual is simultaneously engaged in a competing activity, namely an OT (McDaniel and Einstein, 2000). Thus, the OT may compete for attentional resources with the PM task, leading to a decrease in PM performance.

According to the attentional resource allocation theory (Chen and Zhang, 2003), an individual’s attentional resources can be assigned to different tasks simultaneously, but are intrinsically limited. When the OT occupies excessive attentional resources, the resources assigned to the PM task decrease, adversely affecting PM performance. There are two types of attentional resource allocation, namely the “bottom-up” and “top-down” processes.

Bottom-up processes are driven by the objective characteristics of the task; thus, factors such as task difficulty may affect the allocation of attentional resources. When an OT is more difficult, it occupies more attentional resources, thereby causing the resources assigned to the PM task being reduced, which adversely affects an individual’s PM performance (Mahy et al., 2015).

Substantial evidence has revealed that the objective characteristics (e.g., difficulty) of an OT can affect an individual’s PM performance. For example, Norman and Shallice (1986) proposed attention management system theory, which posits that completing PM tasks requires attentional resources and that the difficulty of an OT directly influences PM performance (Khan et al., 2008). Bisiacchi et al. (2009) found that increased OT difficulty could adversely affect PM performance, regardless of age. Researchers investigated the effect of OT absorption on PM performance with a sample of 9- to 10-year-old and 6- to 7-year-old children; the results revealed that the provision of a less absorbing OT was associated with better PM performance (Kliegel et al., 2013). Mahy et al. (2015) explored whether developmental changes in cognitive control underlie improvements in tbPM in a sample of 5-, 7-, 9-, and 11-year-old children. The results indicated that the children performed worse on the OTs and PM tasks in the divided-attention condition (they had to carry out a secondary task) compared to the full-attention condition. Khan et al. (2008) explored the role of cognitive load in PM performance. The low-cognitive load condition involved minimal information processing (solving general knowledge questions), whereas the high-cognitive load condition required deep processing (solving general knowledge questions and listening to a story). The results revealed that OTs and PM tasks compete for attentional resources, leading to a decrease in PM performance. Sun and Zhou (2009) further explored the mechanism of the effect of OT difficulty on PM performance. Their results suggested that an increase in the difficulty of an OT reduces the available attentional resources that can be assigned to a PM task, thus affecting PM performance.

However, some studies suggested that OT difficulty does not affect PM performance. For example, to manipulate OT difficulty, some researchers required participants to rehearse the word “the” during the process of completing an OT. The results revealed that OT difficulty had no effect on PM performance (Meeks et al., 2007).

In top-down attentional resource allocation, individuals can allocate attentional resources according to their motivation for completing a task. When they perceive an OT to be more motivating, they prioritize this task by allocating more attentional resources to it, which can adversely affect PM performance (Mahy et al., 2015).

Studies have shown that increasing an individual’s motivation level can result in them actively allocating additional attentional resources to a specific task, thereby improving their performance of this task (Costa et al., 2015). For example, Einstein et al. (1992) reported that conditions involving high motivational levels greatly improve the capability of 2- to 4-year-old children to complete PM tasks. Furthermore, studies have manipulated individuals’ motivational levels by using instructions; the results also indicate that higher motivation levels markedly improve PM performance (Kvavilashvili and Fisher, 2007; Penningroth et al., 2012; Penningroth and Scott, 2013). Aberle and Kliegel (2010) reported that raising motivational levels through monetary rewards had a marked effect on PM performance in a sample of children who were in the transition phase from kindergarten to preschool. Kliegel et al. (2010) reported that 5-year-olds outperformed 3-year-olds on an ebPM task under low-incentive conditions, but that under high-incentive conditions performance did not differ between age groups.

Although previous studies have explored the effect of OT difficulty and motivation level on PM performance in different age groups, no consensus has been reached.

Moreover, some previous studies have suggested that the influence of OT difficulty and motivation on PM performance is highly likely to differ between preschool children and other age groups. For example, research has indicated that adults can reasonably allocate their attentional resources according to the importance and the difficulty of a task, but that the attentional resource allocation of younger children is more likely to be affected by task attraction (Meeks et al., 2007).

Although many differences may exist between preschool children and other age groups regarding PM, the participants of previous studies have typically been school-aged children or adults, with preschool children involved less often (Kvavilashvili et al., 2008; Kliegel et al., 2013). Thus, the influence of OT difficulty and motivation level on PM performance in preschool children remains uncertain. Involving preschool children in such studies is of great value for furthering research in this field.

## Current Study

Many studies have suggested that being able to estimate the passage of time is important for successful execution of PM tasks (Kerns, 2000; Aberle and Kliegel, 2010; Zinke et al., 2010). However, the development of time perception ability is ongoing throughout childhood (Sylvie, 2012). Many of the children are unable to precisely estimate the passage of time which may decrease the probability to execute the PM task at a specific time-point successfully. The possibility of delayed retrieval diminishes the effects of time perception on PM, and in turn improves children’s PM performance. Therefore, Experiment 1 was conducted to investigate the developmental trajectory of preschool children and occurrence of delayed retrieval in both tbPM and ebPM tasks.

An essential feature of PM tasks is that they must be performed while an individual is absorbed by another task, namely an OT (McDaniel and Einstein, 2000). Some previous studies have suggested that the influence of OT difficulty and motivation on PM performance is highly likely to differ between preschool children and other age groups (Meeks et al., 2007; Kliegel et al., 2010; Penningroth and Scott, 2013). Therefore, we conducted Experiments 2 and 3 to further explore the influence of OT difficulty on children’s PM performance at both low and high motivation levels for completing the OT.

For Experiments 2 and 3, we selected 5-year-old children as participants for the following reasons: some studies have revealed that the PM performance of 5-year-olds is substantially better than that of 3- to 4-year-olds (Guajardo and Best, 2000; Kliegel and Jäger, 2007) strongly suggesting that this period is a crucial stage in the development of PM ability. Moreover, 5- to 6-year-olds are in the transitional period from preschool to school age. Thus, the successful development of PM ability during this period is necessary for their ability to function independently later in life (Kliegel and Martin, 2003; Wang et al., 2006, 2008). Children with poor PM ability are more likely to experience difficulties at primary school when interacting with teachers and peers (McCauley and Levine, 2004; Kayla and David, 2014). Therefore, studying PM during this particular period has critical educational and social implications (Kayla and David, 2014).

## Experiment 1

### Research Purpose and Hypotheses

Experiment 1 was aimed at exploring the developmental trajectory of preschool children’s PM performance and difference in the delayed retrieval ratio between tbPM and ebPM. Previous studies have indicated that PM follows a clear developmental trajectory (e.g., Kerns, 2000; Shum et al., 2008; Mackinlay et al., 2009; Rendell et al., 2009; Zinke et al., 2010) and that an individual’s PM ability has developed to an extent in early childhood (Kvavilashvili et al., 2001; Kliegel and Jäger, 2007; Kretschmer et al., 2014). However, children’s time perception ability is not fully developed in this period, limiting their ability to precisely estimate the passage of time (Sylvie, 2012). Hence, giving young children a larger “window of opportunity” to perform a PM task is reasonable. Additionally, the execution of a tbPM task is a self-initiated process and is largely dependent on an individual’s time perception ability. By contrast, the execution of an ebPM task generally involves evident external cues (Han, 2012). Therefore, delayed retrieval may be more likely to occur in a tbPM task than in an ebPM task. Hence, the following research hypotheses were proposed:

Hypothesis 1: The PM performance of 5-year-old children is better than that of 4-year-olds, and the performance of 4-year-old children is better than that of 3-year-olds.
Hypothesis 2: The ratio of delayed retrieval in tbPM is larger than that in ebPM.

### Methods

#### Participants

The participants in the current study were 120 preschool children enrolled in Heze city, Shandong province, China. A total of 15 children were excluded because they did not complete the task (withdrew midway through the experiment). Thus, the final data analysis involved 105 children, of whom 51 were boys and 54 were girls. In China, children are generally grouped by age in kindergarten. Three groupings have high prevalence: junior class (3-year-old), middle class (4-year-old), and senior class (5-year-old; Wu et al., 2015). The sociodemographic data of the children are presented in **Table 1**. In addition, none of the participants had any developmental, psychiatric, or neurological disorders, and none of them had previously participated in such an experiment. All participants were reimbursed with gifts (about ¥10) after the experiment. The study was approved by the Human Research Ethics Committee of Shandong Normal University. We explained the experimental process to the parents of all the children in detail before the formal experiment and obtained their informed, written consent.

**Table 1:** Sociodemographic data of the children (*n* = 105).

Age groups	*N*	Age (min)	Age (max)	Age (mean)	Age (*SD*)
3-year-old	38	3.00	3.75	3.33	0.22
4-year-old	32	3.92	4.83	4.27	0.23
5- year-old	35	4.83	5.58	5.22	0.23

#### Experimental Design

To test the hypotheses, the experiment adopted a 2 (PM type: tbPM/ebPM) × 3 (Age group: 3-year-olds/4-year-olds/5-year-olds) two-factor between-subjects design. The independent variables were PM type and age group. The dependent variables were PM performance and delayed retrieval ratio. The participants in each age group were randomly assigned to the two PM type conditions.

The current study used an adapted version of the dual-task paradigm proposed by Einstein et al. (1992). In typical laboratory PM paradigms, participants must execute PM tasks at a specific time-point in the course of performing some OTs. For example, participants were asked to press a special key on a key board when a particular target word was encountered (Einstein et al., 1992). Considering the deficiencies of preschool children’s time perception ability, the typical PM paradigm may be unsuitable for them. Therefore, participants were allowed to execute the PM task in a larger window of opportunity (delayed retrieval) in the adapted version of the PM paradigm.

### Tasks

#### Ongoing Task

**FIGURE 1:**
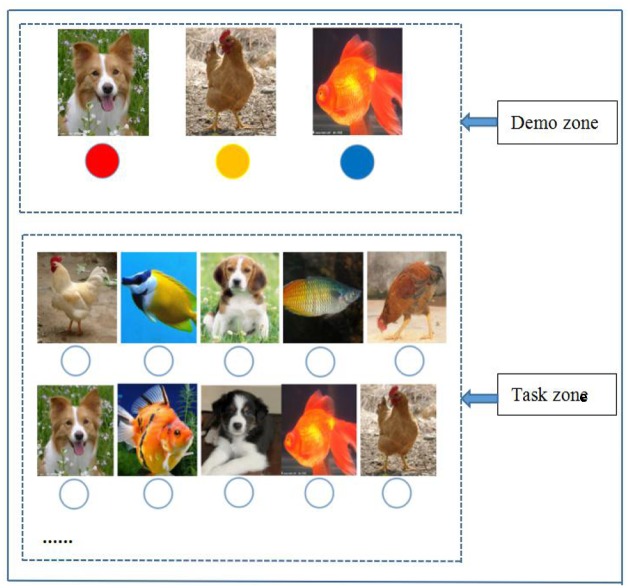
**Paperboard of the easy ongoing task (OT) condition**.

The OT was presented on a paperboard, as shown in **Figure 1**. The figure includes two zones: a demonstration zone and a task zone. In the demonstration zone, three animal–color ball (A–CB) pairs are presented, each of which containing one color paired with one specific animal. In the task zone, a total of 50 animal pictures are presented. A small hole was located under each animal picture. A box of 80 colored balls of three colors (red, yellow, and blue) was made available. Participants were required to place the correct color ball in the hole according to the A–CB pairs. This task was named “arranging rooms for animals” in the instructions. The participants were required to perform the task in a fixed order.

#### PM Tasks

The materials in the tbPM task included a rabbit model, three carrot models, and three hourglasses. These three hourglasses were used to count to 1, 2, and 3 min. The participants were asked to “give” one carrot model to the rabbit model (“feeding a rabbit”) when each hourglass ran out.

The materials in the ebPM task included a dog model (target dog) and three bone models. Similar to the tbPM task, the participants were asked to “give” a bone model to the dog model (“feeding a dog”) when they encountered the target dog in the OT. The pictures of the target dog were displayed on the 10th, 20th, and 30th position in the OT.

#### Procedure

The PM performance of the children was tested in a quiet classroom, and entailed the following specific steps. First, the instructions were presented to the children, who were guided to learn and practice the OT in the experiment. In this step, the children were asked to complete the OT as quickly and precisely as possible. In addition, they were asked to simultaneously complete the PM tasks as soon as the hourglass finished (tbPM task) or a particular picture of a dog was seen (ebPM task). They were instructed that if they failed to perform the PM task at the precise time-point, they could perform it at any time before the next hourglass finished or the next target picture appeared (Han, 2012). Second, the interference task was presented. The interference task involved telling the children a story called “Woodpecker Doctor,” which requires 3 min. Third, the formal experiment was conducted by two experimenters. One of the experimenters monitored the completion status of the OT, and the other filled out the record sheet.

#### Measurement of Dependent Variables

In this study, performance on the OT was evaluated according to two indices: “completion speed” and “completion accuracy.” The completion speed is the ratio of the total number of completed OTs to the time elapsed (unit: task/min). The completion accuracy is the ratio of the correct number to the total number of the OTs completed. In addition, this experiment incorporated two indices, namely the “immediate retrieval” and “delayed retrieval” scores, which were used to evaluate the PM performance of children. For immediate retrieval, the valid retrieval time for tbPM was from 5 s before the hourglass finished until 5 s after the hourglass finished. The valid retrieval time for ebPM was the specific time when the target pictures appeared (Li and Song, 2006). For delayed retrieval, the valid retrieval time of tbPM was the time span from 5 s after the first hourglass finished to 5 s before the subsequent hourglass finished. For ebPM, the valid retrieval time was the time span between the first target picture and the subsequent target picture (Han, 2012). One point was awarded to the participant upon completion of each PM task, with a total of three points.

#### Data Analysis

Data were analyzed using SPSS 16.0.

### Results

**Table 2** shows the completion speed and accuracy for the OTs, as well as the means (with standard deviations) of the PM task scores in the two types of PM task among the three age groups. We also calculated the DR score ratio by comparing the delayed retrieval score to the total PM score (sum of the immediate and delayed retrieval scores). The DR score ratio was used as an index of the portion of the PM tasks that the participants completed in the form of delayed retrieval among all completed PM tasks. Results indicated that delayed retrieval is a crucial retrieval mode for preschool children.

**Table 2:** Ongoing task (OT) and prospective memory (PM) task performance scores and DR score ratio, stratified by age group (*n* = 105).

Age groups	3-year-olds	4-year-olds	5-year-olds	Total
S-OT	5.01 (0.87)	6.48 (1.37)	9.58 (1.12)	6.98 (2.24)
A-OT	0.81 (0.12)	0.84 (0.11)	0.89 (0.10)	0.84 (0.12)
IR-tbPM	0.10 (0.31)	0.24 (0.44)	0.67 (0.76)	0.33 (0.58)
DR-tbPM	0.55 (0.82)	0.94 (1.09)	1.22 (0.94)	0.89 (0.98)
Total-tb	0.65 (0.81)	1.18 (1.13)	1.89 (1.23)	1.22 (1.16)
DR Ratio-tb (%)	84.62	79.66	64.55	72.95
IR-ebPM	1.17 (1.20)	1.53 (1.36)	1.94 (0.90)	1.54 (1.18)
DR-ebPM	0.22 (0.55)	0.20 (0.41)	0.35 (0.49)	0.26 (0.49)
Total-eb	1.39 (1.38)	1.73 (1.48)	2.30 (0.99)	1.80 (1.32)
DR Ratio-eb (%)	15.83	11.56	15.22	14.44

*S-OT: completion speed of the ongoing task; A-OT: completion accuracy of the ongoing task; IR-tbPM: immediate retrieval score in the tbPM task; DR-tbPM: delayed retrieval score in the tbPM task; IR-ebPM: immediate retrieval score in the ebPM task; DR-ebPM: delayed retrieval score in the ebPM task; Total-tb: sum of the immediate and delayed retrieval scores in tbPM; Total-eb: sum of the immediate and delayed retrieval scores in ebPM; DR Ratio-tb: DR score ratio in the tbPM task; DR Ratio-eb: DR score ratio in the ebPM task*.

To investigate the changes in children’s PM performance associated with different PM types and age groups, we conducted a 2 (PM type: tbPM/ebPM) × 3 (age group: 3-year-olds/4-year-olds/5-year-olds) two-way ANOVA on the total PM score. The results showed that the main effect of the two factors all reached significant levels [*F*(1,105) = 6.03, *p* < 0.05, $\eta_{p}^{2}$ = 0.057; *F*(2,105) = 7.57, *p* < 0.001, $\eta_{p}^{2}$ = 0.133]. The performance in ebPM was better than that in tbPM. The results of the *post hoc* tests for different age groups indicated that the performance of 5-year-old children was better than that of the 4-year-old (*p* < 0.05) and 3-year-old children (*p* < 0.001), whereas no difference was observed between the 4- and 3-year-old children (*p* = 0.125).

Additionally, to further investigate the difference in the delayed retrieval ratio among the different PM types and age groups, we conducted a 2 (PM type: tbPM/ebPM) × 3 (age group: 3-year-olds/4-year-olds/5-year-olds) two-way ANOVA on the delayed retrieval ratio. The results revealed that the main effect of PM types reached a significant level [*F*(1,105) = 28.92, *p* < 0.001, $\eta_{p}^{2}$ = 0.226], indicating that a larger proportion of tbPM than ebPM tasks were performed under the condition of delayed retrieval. The main effect of age groups [*F*(2,105) = 0.68, *p* = 0.509, $\eta_{p}^{2}$ = 0.014] and the interaction between the two variables [*F*(2,105) = 0.11, *p* = 0.898, $\eta_{p}^{2}$ = 0.002] were not significant.

### Discussion

The current study used the adapted version of the dual-task paradigm proposed by Einstein et al. (1992), in which the PM task is embedded into the OTs. The average completion speed of the OT varied from 5.03 to 9.56 task/min, and the completion accuracy varied from 80 to 89%. These results revealed that the difficulty level of these tasks was not beyond the children’s capability, but occupied their attentional resources to an extent. Therefore, both the PM task and OT were adapted to suit preschool children for use in the current study.

This study revealed that the PM ability of preschool children was developed to a degree, and that the PM performance of 5-year-old children was significantly higher than that of the 4- and 3-year-old children; these findings are consistent with some previous studies (Guajardo and Best, 2000; Kliegel and Jäger, 2007). However, the difference between 3- and 4-year-old children regarding PM performance was not significant. Hence, we can hypothesize that senior class (i.e., 5 years of age) may be a crucial period in the development of PM ability.

This study revealed that children were able to perform better when they had the opportunity to execute the PM task later (delayed retrieval). The occurrence of delayed retrieval in tbPM was possibly due to the undeveloped time perception ability of preschool children (Sylvie, 2012). When completing a tbPM task, an automatic connection has to be made between a specific time-point and an intentional behavior; therefore, children’s PM performance strongly depends on their time perception ability (Hu and Feng, 2013). However, the time perception ability of preschool children has not fully developed and they are unable to precisely estimate the passage of time (Sylvie, 2012). Thus, these children experienced difficulty in completing a PM task at a specified time-point.

Additionally, delayed retrieval is an important ebPM retrieval mode for preschool children. The possible reasons are as follows: Evident external cues were always provided in the process of completing the ebPM task; however, these tasks can also be automatically activated without any external cues (Hu and Feng, 2013). For example, when someone is asked to take a message for another person, a PM task can be activated not only at the specific time-point when the person appeared, but also before or after the person’s appearance. Therefore, we can hypothesize that the extent of the development of children’s time perception ability also affects their ebPM performance to an extent. Furthermore, when completing an OT, participants may encounter a stimulus similar to the target cue, which could also activate the ebPM task to a degree. For example, when someone is asked to send a letter when they see a mail box, the mail box is not the only effective external cue to help the individual complete the ebPM task. All stimulus related to the letter, such as post offices, can increase the chance of successfully completing the PM task.

The results also revealed that the ratio of delayed retrieval in tbPM is larger than that in ebPM. This is because the tbPM task was mainly completed without any external cues. Thus, completion of the task was more dependent on the children’s time perception ability, which is not yet fully developed (Sylvie, 2012). However, when children complete ebPM tasks, there are generally external cues at the specific time-point which can help them complete the task successfully (Hu and Feng, 2013).

## Experiment 2

### Research Purpose and Hypothesis

The purpose of Experiment 2 was to explore the influence of OT difficulty on children’s PM performance. The results of Experiment 1 revealed that the 5-year-old participants outperformed the 4- and 3-year-olds in PM, which strongly suggests that this period is a crucial stage in the development of PM ability. In addition, 5-year-old children are in the transitional period from preschool to school age, which is a crucial period for developing their ability to function independently later in primary school. Therefore, 5-year-old children were selected as the participants for Experiment 2. According to the theory of bottom-up attentional resource allocation, individuals allocate their limited attentional resources based on the objective characteristics of tasks (e.g., difficulty). Thus, the PM performance of children may be affected by OT difficulty. OTs that are more difficult occupy more attentional resources and thus may lead to a decrease in PM performance (Mahy et al., 2015). On this basis, we hypothesized that:

Hypothesis: The main effects of OT difficulty will be significant, and PM performance under the easy OT condition will be significantly higher than that under the difficult condition.

### Methods

#### Participants

The participants in the current study were 120 senior class children (5-year-olds) enrolled in Heze city, Shandong province, China. A total of 17 children were excluded because they did not complete the task (withdrew midway through the experiment). Thus, 103 children were involved in the final data analysis; 50 were boys and 53 were girls. The mean age of the participants was 5.20 years (*n* = 103, *SD* = 0.16 years) and ranged from 4.83 to 5.58 years. In addition, none of the participants had any developmental, psychiatric, or neurological disorders, and none of them had previously participated in such an experiment. All participants were reimbursed with gifts after the experiment. The study was approved by the Human Research Ethics Committee of Shandong Normal University. We explained the experimental process to the parents of all the children in detail before the formal experiment and all of them provided their informed, written consent.

#### Experimental Design

To test the hypothesis, the experiment adopted a 2 (PM type: tbPM/ebPM) × 2 (OT difficulty: easy/difficult) two-factor between-subjects design. The independent variables were PM type and OT difficulty. The dependent variable was PM performance. The participants were randomly assigned into each experimental condition.

#### OTs and PM Tasks

**FIGURE 2:**
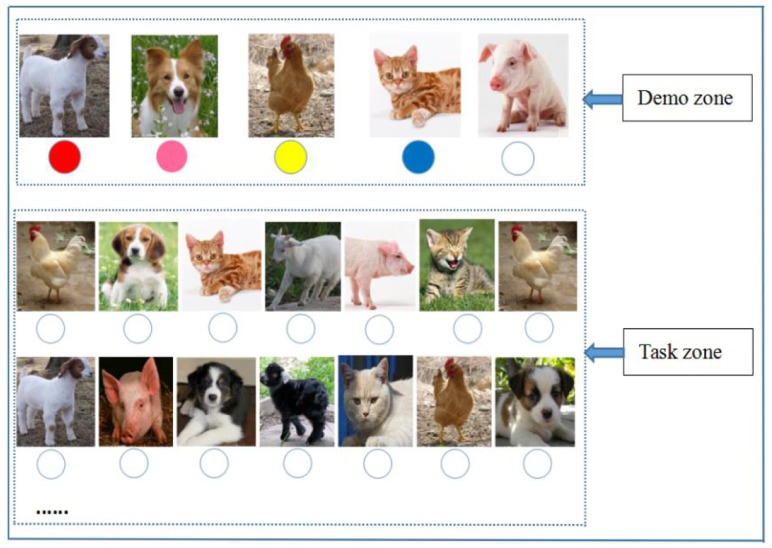
**Paperboard of the difficult OT condition**.

Ongoing task difficulty was manipulated by the number of A–CB pairs. Under the easy OT condition which was applied in Experiment 1, three A–CB pairs were displayed in the demonstration zone (see **Figure 1**). The difficult OT condition was applied in Experiment 2, in which a more complex paperboard with five A–CB pairs was employed (see **Figure 2**) and another box of 80 colored balls (including five colors: red, pink, yellow, blue, and white) was added for this experiment. Other experimental materials for the OTs and PM tasks were identical to those in Experiment 1.

#### Procedure

The procedure was identical to that employed for Experiment 1.

#### Measurement of the Dependent Variables

Because a certain ratio of PM tasks was performed under delayed retrieval conditions, we used the sum of the immediate and delayed retrieval scores to evaluate the participants’ PM performance. The measurement of OT performance was identical to that in Experiment 1.

#### Data Analysis

Data were analyzed using SPSS 16.0.

### Results

**Table 3** shows the participants’ completion speed and accuracy for the OTs. Independent sample *t*-tests revealed significant differences between the easy and difficult OTs regarding both the completion speed and accuracy (*t* = 7.22, *p* < 0.001; *t* = 3.67, *p* < 0.001). Specifically, the completion speed was significantly shorter and the accuracy was significantly higher than those under the difficult condition. **Table 3** also shows the means (with standard deviations) of the participants’ PM performance. Furthermore, we conducted a 2 (PM type: tbPM/ebPM) × 2 (OT difficulty: easy/difficult) two-way ANOVA on the total PM score. The results showed that the main effect of PM type on PM performance was significant [*F*(1,103) = 4.85, *p* < 0.05, $\eta_{p}^{2}$ = 0.047], and that the performance in ebPM was better than that in tbPM. However, the main effect of OT difficulty [*F*(1,103) = 0.44, *p* = 0.507, $\eta_{p}^{2}$ = 0.004] was nonsignificant, which did not support the hypothesis. The interaction between PM type and OT difficulty [*F*(1,103) = 0.17, *p* = 0.683, $\eta_{p}^{2}$ = 0.002] was also nonsignificant.

**Table 3:** Performance of OTs and PM tasks (*n* = 103).

	Easy task	Difficult task
S-OT	9.73 (1.64)	7.53 (1.65)
A-OT	0.88 (0.14)	0.78 (0.14)
Total-tb	2.00 (0.72)	1.80 (1.03)
Total-eb	2.33 (0.96)	2.29 (1.01)

*S-OT: completion speed of the ongoing task; A-OT: completion accuracy of the ongoing task; Total-tb: sum of the immediate and delayed retrieval scores in tbPM; Total-eb: sum of the immediate and delayed retrieval scores in ebPM*.

### Discussion

Experiment 2 was conducted to investigate the influence of OT difficulty on children’s PM performance. Results indicated that when the children performed the difficult OT, their completion speed was longer and their accuracy was lower, indicating significant differences between the easy and difficult tasks regarding the level of difficulty. Therefore, these results demonstrated that the experimental manipulation of OT difficulty was successful.

For this experiment, the proposed hypothesis was based on the theory of bottom-up attentional resource allocation, which suggests that the objective characteristics (e.g., difficulty) of an OT can affect children’s PM performance (Mahy et al., 2015). However, this suggestion was not verified in the experiment: the influence of OT difficulty on both tbPM and ebPM was nonsignificant. This result is not consistent with some previous studies involving participants of other age groups (Khan et al., 2008; Shayne et al., 2008; Bisiacchi et al., 2009; Sun and Zhou, 2009). There are several possible reasons. A lack of sufficient attentional resource capacity may have caused several other factors to determine the allocation of attentional resources. This is despite the OTs and the PM tasks both occupying attentional resources. The motivation level of the participants to complete the task may have played an important role in this process. Specifically, for participants to actively allocate more attentional resources to the task they had to perceive the task’s importance (Einstein et al., 1992, 1997; Kvavilashvili and Fisher, 2007; Aberle and Kliegel, 2010; Penningroth et al., 2012).

According to this analysis, this result might be attributable to the children believing that the PM task was more important, which increased their motivation to complete it. If this is the case, increasing the motivation level for completing the OT may contribute to the appearance of the “difficulty effect” (i.e., the effect of OT difficulty on PM performance). To further explore, the specific reasons that caused the results in Experiment 2, the following experiment was designed.

## Experiment 3

### Research Purpose and Hypothesis

The purpose of Experiment 3 was to investigate the effect of OT difficulty on children’s PM performance in the context of increasing the children’s motivation to complete the OT. Previous studies have shown that raising people’s motivational levels can cause them to actively allocate more attentional resources to a specific task. On this basis, we can infer that if children perceive OT to be crucial, they will prioritize it by allocating more attentional resources to the task; moreover, when the OT is difficult, it occupies more attentional resources, thereby lowering their performance on the PM task. Therefore, we proposed the following hypothesis:

Hypothesis: When increasing participants’ motivation to complete an OT, the main effect of the OT difficulty on PM performance will be significant. PM performance under the easy OT condition is significantly higher than that under the difficult condition.

### Methods

#### Participants

The participants in the current study were 120 senior class children (5-year-olds) enrolled in Heze city, Shandong province, China. A total of 14 children were excluded because they did not complete the task (withdrew midway through the experiment). Thus, 106 children were involved in the final data analysis; 52 were boys and 54 were girls. Specifically, the mean age of the participants was 5.25 years (*n* = 106, *SD* = 0.21 years) and ranged from 4.83 to 5.75 years. In addition, none of the participants had any developmental, psychiatric, or neurological disorders, and none of them had previously participated in such an experiment. All participants were reimbursed with gifts after the experiment. The study was approved by the Human Research Ethics Committee of Shandong Normal University. We explained the experimental process to the parents of all the children in detail before the formal experiment and all of them provided their informed, written consent.

#### Experimental Design

To test the hypothesis, the experiment adopted a 2 (PM type: tbPM/ebPM) × 2 (OT difficulty: easy/difficult) two-factor between-subjects design. The independent variables were PM type and OT difficulty. The dependent variable was PM performance. The participants were randomly assigned to two PM types and OT difficulty conditions.

The difference between the current experiment and Experiment 2 is that we adopted both instruction and material reward to increase the children’s motivation to complete the OT. Specifically, we employed instructions to emphasize the importance of the OT to all of the children, and promised them that they would receive “smiling cartoon badges” as prizes if they performed well in the OT.

#### OTs and PM Tasks

The OTs and PM tasks were identical to those of Experiment 2.

#### Procedure and Measurement of the Dependent Variables

The procedure and measurement were identical to those in Experiment 2.

#### Data Analysis

Data were analyzed using SPSS 16.0.

### Results and Analysis

**Table 4** first shows the participants’ completion speed and accuracy for the OTs. Independent sample *t*-tests revealed a significant difference in the completion speed of the different OTs (*t* = 7.62, *p* < 0.001), indicating that the children completed less tasks per minute when the difficulty of the OT increased. However, no difference was observed in the completion accuracy of the different OTs (*t* = 1.45, *p* = 0.151).

**Table 4:** Performance of OTs and PM tasks (*n* = 106).

	Easy task	Difficult task
S-OT	10.26 (1.50)	7.80 (1.79)
A-OT	0.89 (0.10)	0.86 (0.12)
Total-tb	1.31 (1.01)	0.78 (0.64)
Total-eb	2.12 (0.95)	1.85 (1.23)

*S-OT: completion speed of the ongoing task; A-OT: completion accuracy of the ongoing task; Total-tb: sum of the immediate and delayed retrieval scores in tbPM; Total-eb: sum of the immediate and delayed retrieval scores in ebPM*.

**Table 4** also showed the means (with standard deviations) of PM performance, we conducted a 2 (PM type: tbPM/ebPM) × 2 (OT difficulty: easy/difficult) two-way ANOVA. Results indicated that the main effect of PM type was significant [*F*(1,106) = 24.34, *p* < 0.001, $\eta_{p}^{2}$ = 0.193), and performance in ebPM was higher than that in tbPM. In addition, the main effect of OT difficulty on PM performance was also significant when their motivation to complete the OT was higher [*F*(1,106) = 4.33, *p* < 0.05, $\eta_{p}^{2}$ = 0.041]. PM performance decreased significantly while completing the difficult OT. However, the interaction between PM type and OT difficulty was not significant [*F*(1,106) = 0.49, *p* = 0.487, $\eta_{p}^{2}$ = 0.005].

### Discussion

The results of Experiment 3 revealed that the influence of OT difficulty on children’s PM performance became significant when their motivation to complete the OT was higher. This finding supports the hypothesis for this experiment. These results revealed that the attentional resource allocation of 5-year-old children was affected by not only the objective characteristics of the OT but also by their motivation to accomplish a task. When the motivation level to complete an OT is high, children preferentially allocate more attentional resources to the task. If the OT is more difficult, it will occupy more attentional resources. Thus, insufficient attentional resources are available to allocate toward the PM task, thereby adversely affecting the PM performance. According to this analysis, we can hypothesize that the process of attentional resource allocation in PM is not purely a top-down or bottom-up process; rather, it may be an organic combination of the two processes. Both the motivation of the children and the difficulty of the task play crucial roles in the process of attentional resource allocation.

## General Discussion

Early childhood is an important period in the emergence and development of an individual’s PM ability (Guajardo and Best, 2000; Kliegel and Jäger, 2007). Therefore, the current study first focused on a group of preschool children and investigated the developmental trajectory of their PM ability. The results revealed that preschool children’s PM ability is developed to an extent, which is consistent with previous studies (Kvavilashvili et al., 2001; Rendell et al., 2009; Aberle and Kliegel, 2010). In addition, this study discovered that the PM performance of 5-year-old children was significantly higher than that of the 4- and 3-year-old children, whereas the difference between the 4- and 3-year-old children was not significant. Thus, we can infer that 5-year-old period is an important period in the development of PM ability.

In typical experimental designs, PM tasks generally have to be executed at a specific time-point in the future, which is not consistent with some real-world PM tasks (Han, 2012). Therefore, the present study proposed the concept of delayed retrieval and explored its occurrence in tbPM and ebPM tasks. The results revealed that preschool children were able to performed better when they had the opportunity to execute the PM task later, regardless of their ages. This result suggests that delayed retrieval is an important PM retrieval mode in early childhood. Many issues regarding preschool children’s PM (e.g., the influence of PM task characteristics on PM performance) can be investigated in the context of delayed retrieval in future.

Real-world PM tasks often require that we remember to perform a PM task while we are busily absorbed by an OT (McDaniel and Einstein, 2000). Thus, PM performance may be influenced by the characteristics (e.g., difficulty) of OT. Therefore, Experiment 2 was conducted to investigate the influence of OT difficulty on children’s PM performance. However, the hypothesis proposed for Experiment 2 was not supported in a sample of 5-year-old Chinese children. The result of Experiment 2 was not consistent with some previous studies involving participants of other ages (Khan et al., 2008; Shayne et al., 2008; Bisiacchi et al., 2009; Sun and Zhou, 2009).

According to the “bottom-up” and “top-down” attentional resource allocation theory, we hypothesized that it is not only the objective characteristics of OT that determine the allocation of children’s attentional resources. Both the characteristics of the OT and an individual’s motivation level regarding completing this task may play pivotal roles in this process. Specifically, when participants’ attentional resources are insufficient for completing both the OT and the PM task successfully, they allocate more attentional resources to the specific task only when they perceive its importance (Kvavilashvili and Fisher, 2007; Aberle and Kliegel, 2010; Kliegel et al., 2010; Penningroth et al., 2012).

Consequently, Experiment 3 was conducted to further investigate the effect of OT difficulty on PM performance when the motivation level for completing the OT was higher. We increased the children’s motivation to complete the OT by using both instructions and material rewards. The result revealed that high motivation to complete an OT led to the influence of OT difficulty on children’s PM performance reaching a significant level. These results suggest that 5-year-old children’s attentional resource allocation when completing a PM task is an organic combination of both “top-down” and “bottom-up” processes. Typically, 5-year-old children already possess the ability to allocate their attentional resources actively, adjusting the allocation of their attentional resources to different tasks according to the motivation level it engenders, and thereby ensuring that one of the tasks receives sufficient attentional resources for completion.

## Conclusion

These three experiments demonstrated that the PM performance of the 5-year-old children was significantly better than that of the 3- and 4-year-old children. The children were able to perform better when they had the opportunity to execute the PM task later (delayed retrieval), particularly for tbPM. Although no significant effect of OT difficulty on PM performance was observed for a low-motivation level for completing the OT, when the motivation level was increased, the effect of OT difficulty on PM performance became significant. These results provide insight into the mechanism of attentional resource allocation in PM tasks, and have crucial educational and social implications for preschool children in China.

Several limitations of this study are worth noting. First, to investigate the changes in children’s PM performance, the participants were divided into three groups according to their grade levels. However, as their grades increased, both their age and educational levels increased. Thus, determining which factor (age or educational level) improved children’s PM performance was difficult. Second, to address a flaw in the current experimental paradigms and thereby improve the ecological effect of this study, we proposed the concept of delayed retrieval. However, to account for children’s short attention spans (especially those of 3-year-old children), the delayed retrieval window in this study only comprised an additional minute, which was not fully consistent with real-life situations. Thus, the ecological effect could be further improved by extending the duration of the delayed retrieval window in future studies.

Several concerns remain unaddressed: first, Experiment 1 focused on only preschool children. However, previous studies have revealed that children’s time perception ability is still developing when they enter the primary school (Luo, 2011). Hence, delayed retrieval may persist in the PM of primary students; investigating this concern in primary students in the future is necessary. In addition, Experiments 2 and 3 were conducted with a sample involving only 5-year-old children in this study. However, PM ability develops throughout childhood (Kretschmer et al., 2014). Therefore, future studies should explore these issues among 3- and 4-year-old children, as well as primary students. Finally, apart from OT difficulty and motivation level to complete OT, Many other factors may affect children’s PM performance and should be investigated in future studies. For example, focusing on children’s motivation to complete PM tasks may yield methods for improving children’s PM performance.

## Ethics Statement

This study was carried out in accordance with the recom mendations of the Human Research Ethics Committee of Shandong Normal University with written informed consent from all subjects. All subjects gave written informed consent in accordance with the Declaration of Helsinki. The protocol was approved by the Human Research Ethics Committee of Shandong Normal University.

## Author Contributions

P-gH contributed to the initial idea conception, and the writing of manuscript. LH, Y-lB, and F-qG Contributed to the study design, and critical revisions. YT and M-xX helped to complete the data collection and analysis. All authors approved the final version of the manuscript for publication.

## Conflict of Interest Statement

The authors declare that the research was conducted in the absence of any commercial or financial relationships that could be construed as a potential conflict of interest.
